# The Urban Treatment of Surgical Tuberculosis

**Published:** 1903-10-03

**Authors:** 


### THE URBAN TREATMENT OF SURGICAL TUBERCULOSIS.

Mr. Tubby[1] puts in a very strong protest against the treatment of patients suffering from so-called surgical or external tuberculosis in the hospitals of cities and towns. He shows that such cases treated in the country do much better, as a rule, than those who are treated as in-patients at the London hospitals. From this he argues that special rural hospitals should be established for these cases, which should thereafter be excluded from the hospitals situated in London and other large centres of population. In order to discover the comparative merits of urban and rural hospitals for these cases Mr. Tubby has collected statistics from four London children's hospitals—the Belgrave Hospital, the Evelina Hospital, the Paddington Green Hospital, and the Victoria Hospital—and from one country hospital, the Sevenoaks Hospital for Hip Diseases. His investigations disclose a remarkable discrepancy with regard to the results obtained in these institutions, which is accentuated when it is considered that a considerable number of the patients at the Sevenoaks Hospital are sent there from London hospitals after operative treatment has been tried and failed. Thus, in the year 1901, there were admitted to the four urban hospitals mentioned, 218 cases. The average age of these patients was five years and eight months, and they had undergone treatment for 16 months previously. Forty-five had tuberculous glands in the neck, 10 had tuberculosis of the skin, 90 of the joints, and 64 of the bones. The average duration of their stay in hospital was 41 days, or nearly six weeks. On these patients 415 operations were performed, so that, taking the average, about two operations were performed on each patient; and it is recorded that one wretched child was operated upon 18 times, another 12, and another 11. These statistics constitute, Mr. Tubby considers, a very strong indictment of the urban hospital treatment of tubercle. He believes it to be a moot point whether operations on the bones and joints in city hospitals for children are not productive of as much harm as good in the way of further spreading the disease by infecting fresh tissues. However, the result of all these operations was that only 31·2 per cent. of the cases were cured. Turning now to the Sevenoaks Hospital, in the same period under consideration 42 cases were under treatment. The average age of the patients was a little over 6 years. Thirty were cases of disease of joints, 10 of the bones and spine, and 2 of the lymphatic glands. The average duration of stay in the hospital was 10⅓ months. The total number of operations was 3, and the results show 14 cures, 24 relieved, 3 unrelieved, while 1 died of tuberculous disease of the kidney. In other words, although 190 per cent. of operations were done in the London hospitals as compared with 7 per cent. at the Sevenoaks hospital, the results obtained in the latter institution were better than those obtained in London.

The conclusions to be drawn from these results seem fairly obvious.

[1] Practitioner, Sept., 1903.

